# Learning style analysis of traditional Chinese medicine residents in Taiwan: validation of the traditional Chinese version of the index of learning styles

**DOI:** 10.3389/fmed.2026.1872564

**Published:** 2026-06-19

**Authors:** Yi-Chin Lu, Sze-Yuen Yau, Yi-Chi Chen, Jhih-Yun Wu, Pei-Ching Chiang, Yi-Hsun Tsai, Liang-Wei Tseng

**Affiliations:** 1 Division of Chinese Internal Medicine, Department of Traditional Chinese Medicine, Taoyuan Chang Gung Memorial Hospital, Taoyuan, Taiwan; 2 Chang Gung Medical Education Research Centre, Linkou Chang Gung Memorial Hospital, Taoyuan, Taiwan; 3 Department of Obstetrics and Gynecology, Linkou Chang Gung Memorial Hospital, Taoyuan, Taiwan; 4 Department of Traditional Chinese Medicine, Keelung Chang Gung Memorial Hospital, Keelung, Taiwan; 5 Department of Traditional Chinese Medicine, Chiayi Chang Gung Memorial Hospital, Chiayi, Taiwan; 6 Department of Chinese Medicine, Kaohsiung Chang Gung Memorial Hospital, Kaohsiung, Taiwan; 7 Division of Chinese Acupuncture and Traumatology, Department of Traditional Chinese Medicine, Taoyuan Chang Gung Memorial Hospital, Taoyuan, Taiwan

**Keywords:** Index of Learning Styles (ILS), learning style, questionnaire validation, Traditional Chinese Medicine (TCM), Traditional Chinese Version of the Index of Learning Styles (TC-ILS)

## Abstract

**Introduction:**

Traditional Chinese Medicine (TCM) education in Taiwan is transitioning from an apprenticeship model to a standardized residency program. This unique training cohort of TCM residents integrates the knowledge systems of TCM and Western Medicine (WM). To understand this population, we aim to translate, validate, and use the Traditional Chinese version of the Index of Learning Styles (TC-ILS) to profile the learning style distribution of Taiwanese TCM residents.

**Methods:**

A cross-sectional survey recruited 217 TCM residents from the Chang Gung Memorial Hospital system. Psychometric properties were assessed via Confirmatory Factor Analysis (CFA) and Composite Reliability (CR). Learning style profiles and subgroup differences were also analyzed.

**Results:**

Psychometric evaluation of the TC-ILS yielded mixed fit findings: while RMSEA (0.035) suggested small average residuals, CFI (0.730), TLI (0.714), and SRMR (0.136) indicated substantial misfit. Accordingly, the CFA provides only partial support for the hypothesized four-factor structure, consistent with prior ILS reports involving dichotomous items and highly correlated dimensions. Regarding internal consistency, the CR was acceptable for Sensing/Intuitive (0.639) and Visual/Verbal (0.625), but below the 0.6 standard for Active/Reflective (0.461) and Sequential/Global (0.485). The cohort exhibited strong preferences for Sensing (77.42%), Visual (87.56%), Reflective (62.21%), and Global (59.45%) styles.

**Discussion:**

This study translated, adapted, and assessed the TC-ILS for use in this specific TCM resident cohort. The profile demonstrated a distinct tendency toward Visual, Sensing, Reflective, and Global styles. The Global preference differs from the Sequential style typically observed in WM participants and may reflect features of this specific professional training context.

## 1 Introduction

In Taiwan, Traditional Chinese Medicine (TCM) enjoys widespread application and trust (1), with a utilization rate of TCM reaching as high as 30% as of 2023 (2). Rooted in the ancient master-apprentice system, TCM education has historically emphasized hands-on practice, oral instruction, and prolonged clinical experience accumulation. However, following the establishment of modern medical education systems, TCM education has transitioned from the traditional apprenticeship model to standardized undergraduate curriculum and residency training in the past 60 years (3–5). While this transition aims to integrate TCM into the modern medical system, it requires TCM residents to synthesize classical TCM texts, the evidence-based logic of modern medicine, and extensive clinical skills—such as pulse diagnosis, acupuncture, and manipulation (6). This diverse and complex training is expected to shape learning style preferences distinct from those observed in Western Medicine (WM) residents or general medical students. A further complexity is that approximately one-third of Taiwanese TCM physicians hold dual TCM/WM licenses, thereby being simultaneously influenced by two distinct knowledge paradigms, making this cohort particularly unique (7). Consequently, understanding the cognitive characteristics of this new generation of clinical learners is crucial for optimizing the clinical teaching system.

To address this need, this study utilizes the Felder-Soloman Index of Learning Styles (ILS) (8), developed based on Kolb’s Experiential Learning Theory (Kolb, 1984) (9) and the learning style dimensions defined by Felder and Silverman (10). Although the ILS is widely applied across various disciplines, a statistically acceptable Traditional Chinese version remains lacking in the literature. Previous validation attempts reported Cronbach’s alpha values below the generally acceptable cutoff of 0.5 suggested by Felder and Spurlin (11) for the Active/Reflective (0.48) and Sequential/Global (0.41) dimensions (12). This poor reliability necessitated a new translation and rigorous validation. Accordingly, this study recruited TCM residents from the Chang Gung Memorial Hospitals (CGMH), which are one of the largest medical centers offering a large-scale, standardized TCM residency program in Taiwan, to provide insight into the broader TCM resident population. Since many residents are double majors with backgrounds in both TCM and WM, including those who may later transition to WM practice provides a valuable opportunity to explore this unique trainee subset. Overall, this study offers a unique perspective by characterizing an under-profiled but significant cohort.

This study aims to address these research gaps with two specific objectives: first, to conduct a comprehensive psychometric evaluation of the Traditional Chinese Version of the Index of Learning Styles (TC-ILS), specifically evaluating its construct validity and internal consistency reliability within the Taiwanese TCM resident population; second, to profile and analyze the learning style distribution among Taiwanese TCM residents, thereby contributing to a better understanding of this unique cohort.

## 2 Methods

### 2.1 Study design and participants

This study employed a cross-sectional survey design to examine the structural validity and reliability of the TC-ILS in a cohort of TCM residents in Taiwan. The target population comprised residents from all branches of the CGMH. These include the Northern branches (comprising the Taoyuan, Linkou, and Taipei branches, which serve as joint training units) and the Keelung branch, both located in northern Taiwan; the Chiayi branch in central Taiwan; and the Kaohsiung branch in southern Taiwan. The CGMH system operates one of the few large-scale, systematized TCM residency programs worldwide.

Given the limited national pool of TCM residents and the heterogeneity associated with the profession’s distinctive double major structure, purposive sampling was used to include residents across all training levels, regardless of whether they held a single or double major background. Residents were eligible if they had completed or were undertaking TCM residency training within the five-year period from 1 September 2018 to 31 December 2023, corresponding to postgraduate training levels Resident Year 1 through Resident Year 4 (R1–R4). This sampling window was selected to include individuals with recent, relevant training experiences while preserving recall accuracy and cohort comparability. All participants provided informed consent.

We chose to study TCM residents rather than students primarily because residency training provides a clearer distinction from general medical education groups. This is critical given the dual-track undergraduate programs often seen in Taiwan, where a portion of graduates may eventually pursue careers in WM. By focusing on residents who are actively engaged in postgraduate TCM training, we effectively screen for the population with a primary commitment to TCM practice or at least those who demonstrated a definitive commitment during their clinical specialization phase, even if they later transition to other paths. This method ensures that the analyzed learning style profile accurately reflects the cognitive characteristics of a dedicated TCM professional cohort, rather than a heterogeneous group still undergoing mixed undergraduate education.

### 2.2 Instrument: traditional Chinese version of the index of learning styles

The study used the TC-ILS (Supplementary File S1), which is consistent with the original ILS, comprising 44 forced-choice items, measuring preferences along four bipolar learning dimensions (13):

1. Active–Reflective (ACT/REF): Active: Learns best by doing or discussing. Reflective: Learns best by thinking quietly first.
2. Sensing–Intuitive (SEN/INT): Sensing: Likes facts and established methods. Intuitive: Likes innovation and discovering possibilities.
3. Visual–Verbal (VIS/VRB): Visual: Remembers best what they see (pictures, diagrams). Verbal: Gets more out of words (written or spoken).
4. Sequential–Global (SEQ/GLO): Sequential: Learns in logical, linear steps. Global: Learns in large jumps to get the “big picture.”

Each dimension comprises 11 forced-choice items, yielding a score range from −11 to +11 in increments of 2 (e.g., −11, −9, …, 9, 11). The absolute value of the score reflects preference intensity: scores of 1–3 indicate a balanced preference; 5–7 suggest a moderate preference, meaning learning is smoother in a supportive environment; and 9–11 indicate a strong preference, where significant learning difficulties may arise in a non-supportive environment.

### 2.3 Translation and cultural adaptation

The original ILS was first translated into Traditional Chinese by bilingual experts familiar with both medical education and TCM terminology. An independent professional translator then performed a back-translation to verify semantic consistency with the original English version. Subsequently, a panel of medical education scholars and senior TCM clinicians reviewed the translated items to evaluate their cultural relevance and clarity. The draft version underwent pilot testing with a group of TCM residents, and feedback from this stage informed several iterative revisions to improve item comprehensibility and contextual fit. Finally, the revised instrument was shared with the original ILS authors for verification. This multi-step process ensured that the Traditional Chinese version achieved both semantic equivalence and cultural appropriateness for use within the unique cognitive, clinical, and educational environment of TCM residency training.

### 2.4 Data collection procedures and ethical considerations

Data were collected using an online survey administered through Google Forms, allowing participants to complete the questionnaire at their convenience while ensuring consistency in administration and minimizing data-entry errors. The data collection period spanned from 31 August to 31 December 2023. Before accessing the survey, all participants were presented with detailed information regarding the study purpose, procedures, voluntary nature of participation, and data confidentiality. Only individuals who provided electronic consent were able to proceed with the survey, ensuring that informed consent was both obtained and documented.

The study complied with all ethical guidelines governing research involving human participants. Ethical approval was granted by the Institutional Review Board of the Chang Gung Medical Foundation (IRB No. 202301120B0; approval date: 28 August 2023). All procedures adhered to the principles of the Declaration of Helsinki and institutional regulations for the protection of research participants. No identifiable personal information was collected, and all responses were anonymized prior to analysis to safeguard participant privacy.

### 2.5 Data analysis and statistics

Data analysis proceeded in three stages: (1) descriptive analysis of participant characteristics, (2) psychometric evaluation of the Traditional Chinese ILS using confirmatory factor analysis (CFA), and (3) examination of learning style distributions across the four ILS dimensions. The statistical analysis was performed using R version 4.5.0.

#### 2.5.1 Descriptive statistics

Descriptive statistics were used to summarize participants’ demographic characteristics, including age, sex, training branch, double major status, and current working department/unit. Continuous variables were reported as means and standard deviations, and categorical variables as frequencies and percentages.

#### 2.5.2 Psychometric evaluation of the index of learning styles

The structural validity of the four-factor ILS model was assessed using CFA. Each of the 44 forced-choice items was specified as a categorical indicator loading on its corresponding latent factor. Given that the ILS questionnaire consists of dichotomous items which do not meet the assumption of continuous normal distribution, the Weighted Least Squares Mean and Variance adjusted estimator was employed. This estimator is widely recognized as the most appropriate choice for modeling categorical data, utilizing polychoric correlations as the input matrix (14).

Model fit was evaluated using a multi-index approach consistent with recommendations for structural equation modeling (14, 15). Key fit indices included the chi-square and degrees-of-freedom (*χ*^2^, df), the root mean square error of approximation (RMSEA; ideal < 0.06), the comparative fit index (CFI), the Tucker–Lewis index (TLI; ideal > 0.95), and the standardized root mean square residual (SRMR; ideal < 0.08). Factor loadings, residuals, and modification indices were examined to evaluate local fit and identify potential areas of model misfit. To further evaluate the internal structure, relationships between latent factors were examined to ensure they aligned with the theoretical framework and to verify the distinctiveness of the four dimensions. Internal consistency reliability was assessed using Composite Reliability (CR), which provides a more accurate estimate of latent construct reliability than Cronbach’s alpha within CFA frameworks (16). The CFA for ordinal items based on polychoric correlations was done using the R package lavaan (17).

#### 2.5.3 Analysis of learning style profiles

Learning style distributions across the four ILS dimensions were analyzed both at the polarity level (e.g., Sensing vs. Intuitive). Subgroup analyses were conducted to examine potential differences in learning style polarity by sex, double major status, training branch, and current working department/unit. Chi-square goodness-of-fit tests were used for the overall polarity distributions. Given the small cell counts in several subgroup categories, subgroup comparisons were conducted using Fisher’s exact tests rather than chi-square tests and interpreted as exploratory analyses. A significance level of *p* < 0.05 was applied for all statistical tests.

## 3 Results

### 3.1 Participant demographics

A total of 217 TCM residents completed the questionnaire. The sex distribution showed a female predominance (127 females, 58.53%, 90 males, 41.47%), with a mean age of 32.41 ± 4.04 years. Nearly half of the participants were double majors (97, 44.70%). Regarding training locations, the Northern branches represented the largest group (105 participants, 48.39%), followed by Kaohsiung (68, 31.34%), Keelung (24, 11.06%), and Chiayi (20, 9.22%). In terms of current working department/unit, the majority were still serving in medical centers (125, 57.60%), followed by local clinics (79, 36.41%). A small number were working in regional hospitals (6, 2.76%) or had transitioned to WM practice (4, 1.84%). The three participants listed as unemployed were not affiliated with any clinical department/unit at the time of the survey and were not in a short-term transition to another clinical position. Table 1 summarizes the demographic characteristics.

**Table 1:** Demographic characteristics of surveyed participants (*N* = 217).

Characteristics	Participants number (%)
Sex	
Female	127 (58.53)
Male	90 (41.47)
Age (years), Mean ± SD: 32.41 ± 4.04	
25–29	61 (28.11)
30–34	107 (49.31)
35–39	40 (18.43)
40–44	5 (2.30)
45–49	3 (1.38)
50–54	1 (0.46)
Double major (WM/TCM)	
Yes	97 (44.70)
No (TCM only)	120 (55.30)
CGMH training branch	
Northern (Linkou/Taoyuan/Taipei)	105 (48.39)
Kaohsiung	68 (31.34)
Keelung	24 (11.06)
Chiayi	20 (9.22)
Current working department/unit	
Medical center (TCM)	125 (57.60)
Regional hospital (TCM)	6 (2.76)
Local clinic (TCM)	79 (36.41)
Unemployed	3 (1.38)
Medical center (WM)	2 (0.92)
Regional hospital (WM)	2 (0.92)

TCM, Traditional Chinese Medicine, WM, Western Medicine, CGMH, Chang Gung Memorial Hospital.

### 3.2 Psychometric validation of the traditional Chinese version of the index of learning styles

**Figure 1:**
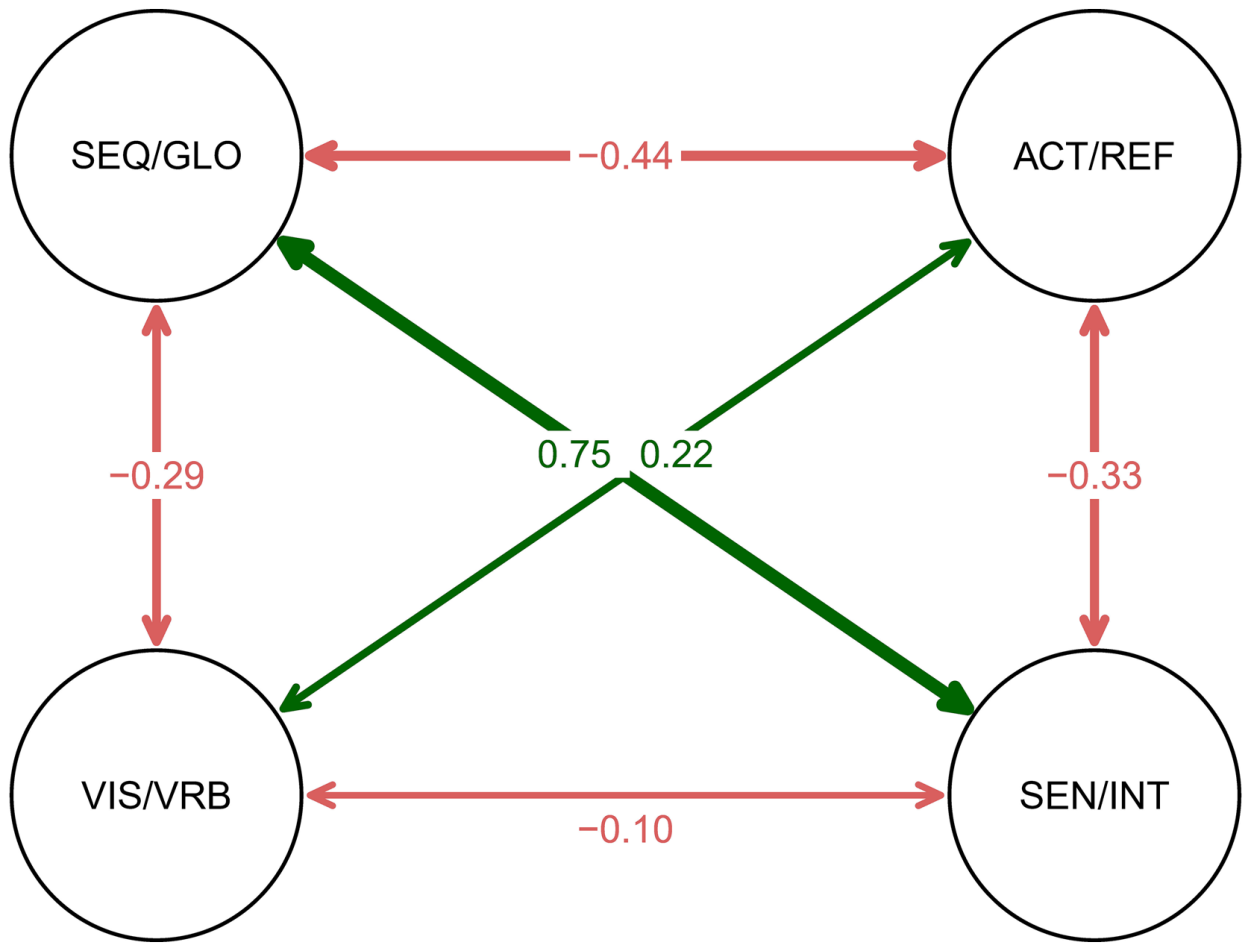
Latent factor correlations of the four-factor CFA model of the TC-ILS (*N* = 217). Circles represent the four latent dimensions. Double-headed arrows indicate standardized latent factor correlations, with line thickness reflecting correlation magnitude. Green arrows indicate positive correlations, and red arrows indicate negative correlations. ACT/REF, Active/Reflective; SEN/INT, Sensing/Intuitive; VIS/VRB, Visual/Verbal; SEQ/GLO, Sequential/Global; CFA, Confirmatory Factor Analysis; TC-ILS, Traditional Chinese Version of the Index of Learning Styles.

The results of the CFA for the TC-ILS four-factor model indicated mixed model fit findings. The RMSEA (0.035) was excellent, suggesting small residuals and a good fit of the model based on this index. However, the CFI (0.730) and TLI (0.714) were both lower than the acceptable 0.95 threshold, and the SRMR (0.136) exceeded the ideal value of 0.08. The latent factor correlations are presented in Figure 1, and item-level standardized factor loadings are provided in Supplementary Table S2.

Regarding latent factor reliability, the CR for the ACT/REF (0.461) and SEQ/GLO (0.485) dimensions did not reach the generally accepted standard of 0.60. The Sensing/Intuitive (0.639) and Visual/Verbal (0.625) dimensions achieved only marginally acceptable levels (Table 2). Furthermore, the latent factor correlation analysis and modification indices revealed a significantly high positive correlation (0.755) between the SEN/INT and SEQ/GLO factors.

**Table 2:** Summary of four-factor CFA model structural parameters.

Parameter category	Parameter name	Value (scaled)
A. Overall model fit indices	*χ* ^2^	1937.48
	df	946
	*χ*^2^/df	1.263
	*p*	< 0.001
	RMSEA	0.035
	RMSEA 90% C.I.	[0.028, 0.041]
	CFI	0.730
	TLI	0.714
	SRMR	0.136
B. Factor reliability (CR)	ACT/REF	0.461
	SEN/INT	0.639
	VIS/VRB	0.625
	SEQ/GLO	0.485
C. Latent factor correlations (Std.)	SEN/INT ∼ SEQ/GLO	0.755
	ACT/REF ∼ SEQ/GLO	−0.444
	ACT/REF ∼ SEN/INT	−0.330
	VIS/VRB ∼ SEQ/GLO	−0.292
	ACT/REF ∼ VIS/VRB	0.220
	SEN/INT ∼ VIS/VRB	−0.098

*χ*^2^, Chi-Square; df, Degrees of Freedom; RMSEA, Root Mean Square Error of Approximation; CFI, Comparative Fit Index; TLI, Tucker–Lewis Index; SRMR, Standardized Root Mean Square Residual; CR, Composite Reliability; CFA, Confirmatory Factor Analysis; ACT, Active; REF, Reflective; SEN, Sensing; INT, Intuitive; VIS, Visual; VRB, Verbal; SEQ, Sequential; GLO, Global.

### 3.3 Learning style profile distribution and subgroup analysis

**Figure 2:**
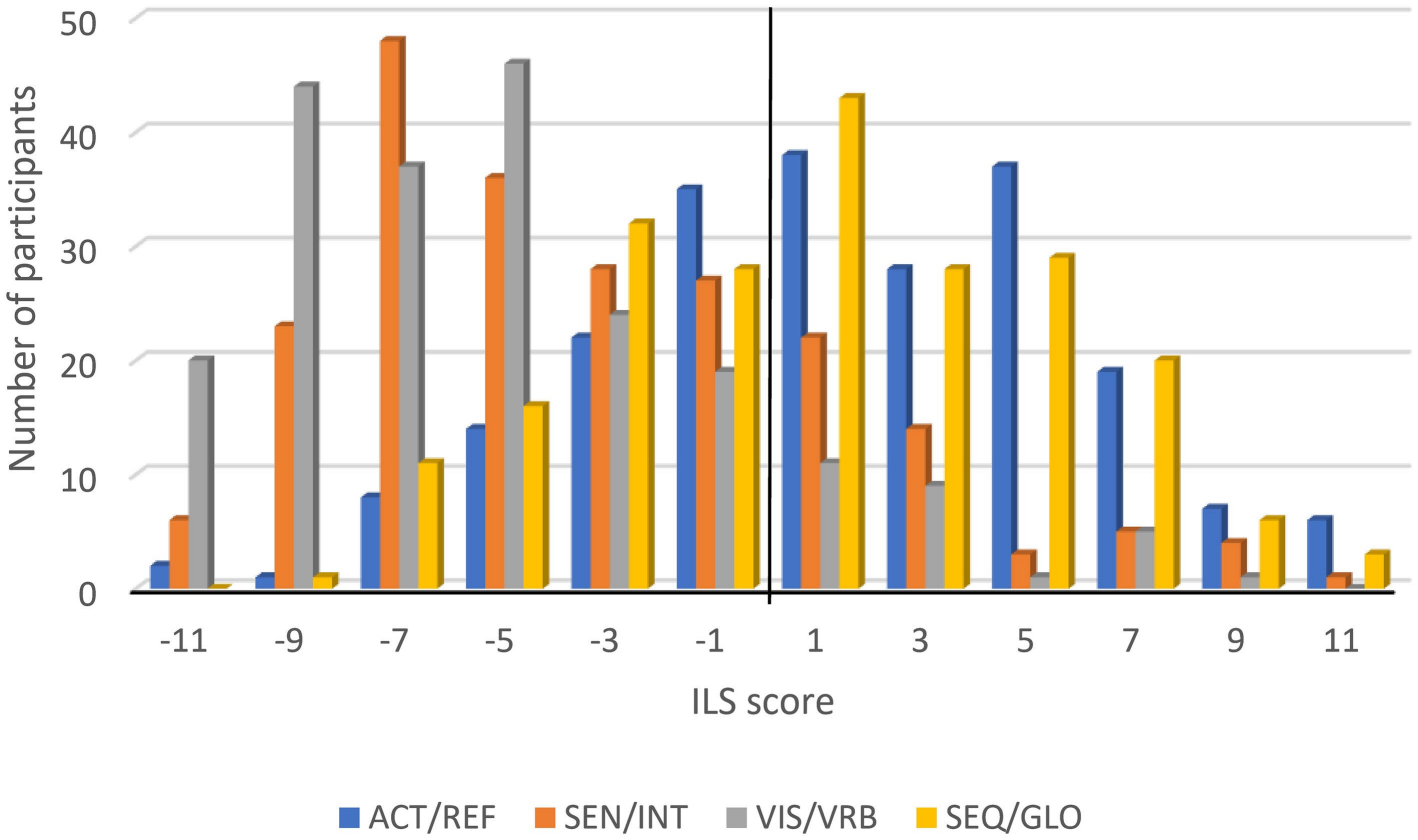
Grouped bar chart of participants’ ILS score distributions across four dimensions. ACT/REF, Active/Reflective; SEN/INT, Sensing/Intuitive; VIS/VRB, Visual/Verbal; SEQ/GLO, Sequential/Global; ILS, Index of Learning Styles.

The learning style distribution among TCM residents showed significant tendencies (*p* < 0.01 across all dimensions), indicating pronounced preferences that diverge sharply from a balanced distribution. The most extreme preference was observed in the VIS/VRB dimension, with an overwhelming 87.56% (190/217) of participants favoring the Visual style. A significant skew was also present in the SEN/INT dimension, where 77.42% (168/217) preferred Sensing. Furthermore, more than half of the residents favored the Reflective and Global approaches: 62.21% (135/217) preferred the Reflective style over the Active style, and 59.45% (129/217) preferred the Global style over the Sequential style. This distribution, summarized in Table 3 and visualized in Figure 2, showed a marked predominance of Visual, Sensing, Reflective, and Global preferences within this cohort.

**Table 3:** Learning style preference distribution by polarity of participants (N = 217).

Dimension	Participants no. (%)
ACT/REF	
ACT	82 (37.79)
REF	135 (62.21)
*p*-value	< 0.001
SEN/INT	
SEN	168 (77.42)
INT	49 (22.58)
p-value	< 0.001
VIS/VRB	
VIS	190 (87.56)
VRB	27 (12.44)
p-value	< 0.001
SEQ/GLO	
SEQ	88 (40.55)
GLO	129 (59.45)
p-value	= 0.005

ACT, Active; REF, Reflective; SEN, Sensing; INT, Intuitive; VIS, Visual; VRB, Verbal; SEQ, Sequential; GLO, Global.

In the exploratory subgroup analyses using Fisher’s exact tests, no statistically significant differences in learning style polarities were observed across sex, double major status, CGMH training branch, or current working department/unit (*p* > 0.05 for all comparisons). Table 4 provides the detailed subgroup analysis results.

**Table 4:** Learning style preferences by sex, double major status, training branch, and current working department/unit [No. (%)].

Dimension	Sex		Double major (WM and TCM)		CGMH training branch				Current working department/unit					
	Male	Female	Yes	No (TCM only)	Linkou/Taoyuan/Taipei	Kaohsiung	Keelung	Chiayi	Medical center	Regional hospital	Local clinic	Unemployed	Medical center (WM)	Regional hospital (WM)
ACT	31 (34.44)	51 (40.16)	35 (36.08)	47 (39.17)	42 (40.00)	25 (36.76)	7 (29.17)	8 (40.00)	51 (40.80)	1 (16.67)	27 (34.18)	2 (66.67)	0 (0.00)	1 (50.00)
REF	59 (65.56)	76 (59.84)	62 (63.92)	73 (60.83)	63 (60.00)	43 (63.24)	17 (70.83)	12 (60.00)	74 (59.20)	5 (83.33)	52 (65.82)	1 (33.33)	2 (100.00)	1 (50.00)
*p*	0.478		0.674		0.795				0.518					
SEN	65 (72.22)	103 (81.10)	74 (76.29)	94 (78.33)	80 (76.19)	51 (75.00)	19 (79.17)	18 (90.00)	95 (76.00)	4 (66.67)	64 (81.01)	2 (66.67)	1 (50.00)	2 (100.00)
INT	25 (27.78)	24 (18.90)	23 (23.71)	26 (21.67)	25 (23.81)	17 (25.00)	5 (20.83)	2 (10.00)	30 (24.00)	2 (33.33)	15 (18.99)	1 (33.33)	1 (50.00)	0 (0.00)
*p*	0.140		0.746		0.574				0.556					
VIS	82 (91.11)	108 (85.04)	83 (85.57)	107 (89.17)	93 (88.57)	58 (85.29)	20 (83.33)	19 (95.00)	106 (84.80)	5 (83.33)	73 (92.41)	3 (100.00)	1 (50.00)	2 (100.00)
VRB	8 (8.89)	19 (14.96)	14 (14.43)	13 (10.83)	12 (11.43)	10 (14.71)	4 (16.67)	1 (5.00)	19 (15.20)	1 (16.67)	6 (7.59)	0 (0.00)	1 (50.00)	0 (0.00)
*p*	0.214		0.536		0.621				0.241					
SEQ	33 (36.67)	55 (43.31)	40 (41.24)	48 (40.00)	35 (33.33)	35 (51.47)	11 (45.83)	7 (35.00)	47 (37.60)	2 (33.33)	37 (46.80)	1 (33.33)	0 (0.00)	1 (50.00)
GLO	57 (63.33)	72 (56.69)	57 (58.76)	72 (60.00)	70 (66.67)	33 (48.53)	13 (54.17)	13 (65.00)	78 (62.40)	4 (66.67)	42 (53.20)	2 (66.67)	2 (100.00)	1 (50.00)
*p*	0.400		0.890		0.103				0.689					

TCM, Traditional Chinese Medicine, WM, Western Medicine, CGMH, Chang Gung Memorial Hospital, ACT, Active, REF, Reflective, SEN, Sensing, INT, Intuitive, VIS, Visual, VRB, Verbal, SEQ, Sequential, GLO, Global.

## 4 Discussion

### 4.1 Psychometric evaluation of the TC-ILS: construct validity and internal consistency

Regarding the construct validity of the TC-ILS, the CFA revealed a complex profile: while the RMSEA (0.035) suggested small average residuals, the CFI (0.730), TLI (0.714), and SRMR (0.136) indicated substantial misfit. Accordingly, the CFA provides only partial support for the hypothesized four-factor structure, a finding consistent with prior reports of the ILS such as a previous CFA conducted on nursing students using the original instrument (CFI = 0.779, TLI = 0.766, and RMSEA = 0.034) (18). The near-identical nature of these fit indices suggests that the TC-ILS possesses construct validity similar to the original instrument, particularly when applied to dichotomous items and highly correlated dimensions. These fit indices are particularly sensitive to inter-factor correlations, which likely explains the low CFI/TLI scores, especially given the high correlation between the SEN/INT and SEQ/GLO factors (0.755), as shown in Table 2. Given this high correlation, we further examined discriminant validity using the Heterotrait-Monotrait Ratio. The resulting value of 0.857 remained below the 0.90 threshold (19), suggesting that while these dimensions are highly interrelated, they maintain adequate discriminant validity within this cohort. This pattern is theoretically consistent with Felder and Spurlin’s explanation that sequential learners, who build understanding in logical steps, are generally more likely to be sensing, whereas global learners, who grasp concepts holistically, are more likely to be intuitive (11). In the present TCM resident cohort, the SEN/INT–SEQ/GLO association may also reflect the dual demands of concrete clinical information gathering and holistic diagnostic reasoning, in which observable signs and symptoms are integrated into broader syndrome patterns (20, 21).

In this study, internal consistency was assessed using CR. The SEN/INT (0.639) and VIS/VRB (0.625) dimensions achieved acceptable levels, meeting the generally accepted standard of 0.60. However, the CR values for the ACT/REF (0.461) and SEQ/GLO (0.485) dimensions were low, indicating notable measurement error at the latent level; findings for these dimensions should be interpreted as descriptive preferences rather than precise trait estimates. Although scholars recommend CR as a more robust alternative to Cronbach’s alpha, the practical difference is often minimal (22). For comparison, and considering that previous ILS studies commonly report Cronbach’s alpha due to the instrument’s unique nature, the calculated Cronbach’s alpha values were: ACT/REF: 0.567, SEN/INT: 0.644, VIS/VRB: 0.619, and SEQ/GLO: 0.515. Felder and Spurlin in their 2005 publication suggested that a value of 0.5 is a reasonable standard for the ILS, as it is a measure of attitudes rather than stable personality traits (11). In this context, the reliability of the instrument is acceptable, with all values falling within the 0.5 to 0.7 range, consistent with previous research (11, 23). The lower Cronbach’s alpha for the ACT/REF (0.567) and SEQ/GLO (0.515) dimensions also aligns with trends observed in prior ILS literature (11, 23). In summary, the TC-ILS translated and utilized in this study meets the commonly reported psychometric standards for the ILS, with known limitations in model fit and internal consistency, and is suitable for use in subsequent research focused on cohort-level profiling and descriptive analyses.

### 4.2 Profiling the unique learning style of TCM residents

The core finding of this study is that TCM residents exhibit strong preferences in their learning styles: they overwhelmingly favor the Visual style (87.56%) and the Sensing style (77.42%). Furthermore, a majority also preferred the Reflective style (62.21%) and the Global style (59.45%). This profile is inconsistent with prior research, which found that medical students worldwide generally display Visual, Sensing, and Sequential characteristics, with variation in the ACT/REF dimension (24–27). Dental residents showed a similar trend (28). In contrast, the TCM resident cohort showed a different pattern, most notably a Global tendency, which differs from the Sequential profile commonly reported among general medical students. This finding is particularly interesting because, theoretically, individuals who prefer Sensing are more likely to be Sequential learners. This is supported by previous research on medical and dental students (24–28), validation studies of the ILS (11), and the high correlation between the SEN/INT and SEQ/GLO factors found in this study. Therefore, the coexistence of a strong Sensing preference and a Global tendency in this cohort warrants further investigation. Importantly, although nearly half of the participants were double majors (44.7%), and not all worked in medical centers, exploratory subgroup analyses showed no statistically significant differences in learning styles across double major status or current working department/unit. This pattern may therefore characterize the present TCM resident cohort, although further validation in broader samples is needed.

When interpreting these results, it is important to remember that learning styles are not always fixed. They can be shaped by teaching methods or different environments (29). In many past studies (24–28, 30), medical and other health profession students consistently showed a dominant preference for Sensing and Sequential styles. This tendency may reflect both the nature of the medical discipline and the result of long-term training. Preclinical courses are filled with foundational facts that require memory and precision (31, 32), which fits the Sensing style. TCM education is no exception. In Taiwan, the TCM curriculum covers both TCM and WM, meaning the amount of basic knowledge students must master is even larger. It is likely that students who already prefer Sensing find this process easier, and residents who have completed this training may have become more accustomed to this style.

The main difference between TCM residents and other health professions lies in the Sequential versus Global dimension. In most medical fields, Sequential learning or practice is the default setting. Most courses follow a sequence of increasing difficulty (30). For example, students must master physiology before learning pathology and clinical treatment. Additionally, under evidence-based medicine, standardized protocols and guidelines are the most respected clinical logics (33), which may reinforce the Sequential style. In contrast, the Global tendency among TCM residents may be related to the holistic philosophy of TCM (21, 34). TCM views the human body as a whole. Its core concepts are “Qi, blood, Yin-Yang, Zang-fu, and meridians” rather than single cells or specific organs. In TCM theory, illness is seen as an imbalance of the patient’s overall state. The human body is a whole system where functions may be in or out of balance. Therefore, the goal of diagnosis is to understand the nature of the imbalance, and treatment focuses on restoring harmony (35, 36).

An interesting extension of this finding is that prior research indicated medical students tend to select specialties that match their cognitive styles (37). If this is true, for double major medical students who can choose between TCM and WM, the decision to enter a TCM residency may be driven by a desire to practice medicine under a paradigm that better fits their learning style.

### 4.3 Limitations and future directions

As this is the first study to translate the TC-ILS and achieve the validity and reliability standards established by the original authors, as well as the first study to use this instrument to validate the learning styles of TCM residents, several limitations must be acknowledged. First, the applicability of the TC-ILS is confined to this specific sample (residents within the CGMH), and its cultural generalizability requires broader investigation. Second, although the sample was recruited from different training sites covering northern, central, and southern Taiwan, all sites belong to the same hospital system. Therefore, the results may have limited representativeness and cannot be definitively extrapolated to all TCM residents from other hospitals in Taiwan. Third, this study focused solely on TCM residents and did not include TCM undergraduate students. Fourth, since learning styles can develop and change over time (23, 38, 39), the single cross-sectional design limits our ability to capture the dynamic evolution of these styles. Furthermore, subgroup analyses should be interpreted cautiously because several subgroup categories, particularly CGMH training branch and current working department/unit, contained small numbers of participants. Based on these limitations, future research should consider the following: First, expanding the scope to include similar studies at more higher education institutions across Taiwan. Second, conducting longitudinal analyses to observe and describe the dynamic behavior and changes in learning styles over the course of training.

While the hypothesis that matching teaching styles to learning styles improves educational outcomes has been widely challenged, individuals still reliably express their learning preferences (40–42). Felder in his 2020 article further suggested that instructors should use these preferences to design well-rounded and balanced educational programs that strive to accommodate all student types (13). Therefore, understanding the distribution of trainees’ cognitive preferences is essential for reviewing and optimizing the TCM training environment. Consistent with our aim of using this instrument as a tool for cohort profiling, we believe the findings describe the cohort’s tendencies and provide important insights and references for the improvement of future TCM education.

## 5 Conclusion

This study translated and validated the TC-ILS for TCM residents, providing a tool with acceptable reliability for future research in Taiwan. The analysis showed strong preferences for Visual (87.56%), Sensing (77.42%), Reflective (62.21%), and Global (59.45%) styles. The marked Global tendency differs from the Sequential profile commonly reported among general medical students. This finding may reflect the distinctive educational and clinical context of TCM residency, but it should be validated in future comparative and longitudinal studies.

## Data Availability

The raw data supporting the conclusions of this article will be made available by the authors, without undue reservation.

## Glossary

ACT: Active
CFA: Confirmatory Factor Analysis
CFI: Comparative Fit Index
CGMH: Chang Gung Memorial Hospital
CR: Composite Reliability
*χ* ^2^: Chi-Square
df: Degrees of Freedom
GLO: Global
ILS: Index of Learning Styles
INT: Intuitive
IRB: Institutional Review Board
REF: Reflective
RMSEA: Root Mean Square Error of Approximation
SD: Standard Deviation
SEN: Sensing
SEQ: Sequential
SRMR: Standardized Root Mean Square Residual
TC-ILS: Traditional Chinese Version of the Index of Learning Styles
TCM: Traditional Chinese Medicine
TLI: Tucker–Lewis Index
VIS: Visual
VRB: Verbal
WM: Western Medicine
