# Supplementary material for: Learning style analysis of traditional Chinese medicine residents in Taiwan: validation of the traditional Chinese version of the index of learning styles
Source: Front Med (Lausanne). 2026 Jun 19;13:1872564. doi: 10.3389/fmed.2026.1872564 (PMC13335680; doi:10.3389/fmed.2026.1872564)
Supplement: Supplementary file 1 [file Data_Sheet_1.PDF]

LEARNING STYLE PROFILE OF TRADITIONAL CHINESE MEDICINE (TCM) RESIDENTS IN TAIWAN

1. STUDY COHORT (PARTICIPANTS)

N = 217
TCM RESIDENTS

LOCATED AT HOSPITALS ACROSS TAIWAN

44.7%
HOLD DUAL LICENSES
(DOUBLE MAJOR IN TCM & WESTERN MEDICINE)

2. METHODOLOGY

INSTRUMENT:
TRADITIONAL CHINESE VERSION OF THE INDEX OF LEARNING STYLES (TC-ILS)

SURVEY

FORMAT:
44-ITEM FORCED-CHOICE QUESTIONNAIRE

3. DOMINANT LEARNING STYLES (RESULTS)

STATISTICALLY SIGNIFICANT PREFERENCES OBSERVED IN ALL DIMENSIONS ($P < .01$)

- **VISUAL** (87.56%)
- **SENSING** (77.42%)
- **REFLECTIVE** (62.21%)
- **GLOBAL** (59.45%)

4. CORE INSIGHT & DISCUSSION: THE GLOBAL DIVERGENCE

WM RESIDENTS

SEQUENTIAL LOGIC

TCM RESIDENTS

GLOBAL TENDENCY

→ **HOLISTIC PHILOSOPHY**

INFLUENCE OF TCM'S HOLISTIC VIEW ON COGNITIVE PROCESSING & PROFESSIONAL IDENTITY

→ **GLOBAL THINKING: A HOLISTIC APPROACH**
(Overall Thinking)

5. CONCLUSION & IMPLICATIONS

THESE FINDINGS PROVIDE A VITAL REFERENCE FOR OPTIMIZING TRAINING ENVIRONMENTS TO MATCH TRAINEE **COGNITIVE NEEDS**
