# Supplementary material for: Learning style analysis of traditional Chinese medicine residents in Taiwan: validation of the traditional Chinese version of the index of learning styles
Source: Front Med (Lausanne). 2026 Jun 19;13:1872564. doi: 10.3389/fmed.2026.1872564 (PMC13335680; doi:10.3389/fmed.2026.1872564)
Supplement: Supplementary file 2 [file Data_Sheet_2.pdf]

Traditional Chinese Version of the Index of Learning Styles Questionnaire (TC-ILS)

1. 在我
(a) 實際嘗試一遍後我會更了解某事物。
(b) 仔細想過一遍後我會更了解某事物。

2. 我更想被認為是一個
(a) 注重實際的人。
(b) 追求創新的人。

3. 當我回憶我昨天做了什麼事時，腦中通常會出現
(a) 一個圖片。
(b) 一些字句。

4. 我傾向於
(a) 了解一個主題的細節，但可能對其完整的架構印象比較模糊。
(b) 了解一個主題的整體架構，但可能對細節印象比較模糊。

5. 當我學習新事物時，對我更有幫助的是去
(a) 談論它。
(b) 思考它。

6. 如果我是一名老師，我更想教授一門什麼樣的課程？
(a) 關於事實和現實生活情境。
(b) 關於想法和理論。

7. 我傾向用什麼樣的方式獲得新資訊？
(a) 照片、圖片、圖表或地圖。
(b) 文字指引或口語資訊。

8. 一旦我了解了
(a) 各個拆開的部分，我便能了解完整的事物如何運作。
(b) 完整的事物，我便能了解各個部份如何互相配合。

9. 當我身處學習小組中，要一起研讀、處理困難的內容時，我更傾向於

(a) 參與並貢獻想法。
(b) 靜靜聆聽。

10. 對我來說比較容易的是
(a) 學習事實。
(b) 學習概念。

11. 在一本有豐富圖片與表格的書中，我傾向於
(a) 仔細地看過表格與圖片。
(b) 聚焦在文字內容上。

12. 當我在解數學題時，
(a) 我通常需要嘗試，一步一步循序漸進解出答案。
(b) 我通常直覺知道答案的方向，但要花一些時間想出中間解題的步驟。

13. 在我曾參與過的課堂中，
(a) 我通常會認識很多同學。
(b) 我通常不會認識很多同學。

14. 在閱讀非小說類書籍時，我比較喜歡
(a) 能教我新事實或告訴我如何做某事的內容。
(b) 能刺激我思考新想法的內容。

15. 我比較喜歡
(a) 在黑板上呈現很多圖示的老師。
(b) 花很多時間解釋事物的老師。

16. 當我分析一個故事或一本小說時，
(a) 我會思考其中發生的各個事件，並試著將它們結合在一起以梳理出主題。
(b) 閱畢後我就能明確理解主題，但需要回頭找尋展現出這些主題的事件。

17. 當我開始做一份作業時，我更傾向於
(a) 立刻開始著手解答。
(b) 先試著完全理解問題本身。

18. 我更喜歡
(a) 確定性的概念。

(b) 理論性質的概念。

19. 我更能記得
(a) 我看到的東西。
(b) 我聽到的東西。

20. 對我而言，教師更重要的是
(a) 能夠以清晰的步驟將教學內容呈現出來。
(b) 能夠給我一個整體的概念並將教學內容連結到其他科目。

21. 我更喜歡
(a) 參與學習小組。
(b) 獨自學習。

22. 我更常被認為
(a) 能很謹慎地處理工作細節。
(b) 能用有創意的方式進行工作。

23. 若我要獲得前往新地點的指示時，我更喜歡
(a) 透過地圖。
(b) 透過書面指引。

24. 我學習時
(a) 步調相對穩定。如果我努力學習，最終會理解它。
(b) 進程時而進展，時而停滯。我可能前一刻完全困惑，然後突然一切都豁然開朗。

25. 做一件事時我通常會
(a) 先嘗試看看。
(b) 先想想看我要怎麼做。

26. 當我以娛樂為目的閱讀時，我希望作者
(a) 清楚地表達他們的意思。
(b) 用有創意而有趣的方式表達事物。

27. 當我在課堂上看到一個圖表或素描時，我通常會記得
(a) 那幅圖片。
(b) 老師的解說內容。

28. 在思考大量訊息時，我比較容易會
(a) 關注在細節而忽略整體大局。
(b) 嘗試先理解整體大局再深入細節。

29. 我更容易記得
(a) 我做過的事。
(b) 我思考過很多的事。

30. 當我需要執行一個任務時，我比較喜歡
(a) 專精一個能夠完成它的方法。
(b) 想出不同的新方式來完成它。

31. 當有人要向我展示數據時，我更喜歡的型式是
(a) 表格或圖形。
(b) 總結結果的文字。

32. 當寫一篇論文時，我更有可能
(a) 先著手處理（思考或撰寫）論文的開頭，然後逐步往下進展。
(b) 先處理（思考或撰寫）論文的不同部分，然後再將它們進行排序。

33. 當我必須進行團隊工作時，首先我會
(a) 進行團體腦力激盪，讓每個人貢獻想法。
(b) 進行個別的腦力激盪，統整後再在團隊中比較各自的想法。

34. 我認為被稱作
(a) 「明智的」是更高的讚賞。
(b) 「有想像力的」是更高的讚賞。

35. 當我在派對中認識人們時，我更容易記得
(a) 他們的長相。
(b) 他們怎麼談論自己。

36. 當我學習一個新科目時，我比較喜歡
(a) 專注在這個科目，盡可能多地學習相關知識。
(b) 嘗試連結該科目與其他相關科目。

37. 我更可能被認為是

(a) 外向的。
(b) 內向的。

38. 我比較喜歡強調
(a) 具體內容的課程(事實、數據) 。
(b) 抽象內容的課程(概念、理論) 。

39. 要放鬆娛樂時，我更想要
(a) 看電視。
(b) 閱讀。

40. 有些老師會在上課前提供他們授課內容的大綱，這些大綱對我來說
(a) 有點幫助。
(b) 非常有幫助。

41. 以小組的型式完成作業，並且整個組別共享同一個成績的概念對我來說
(a) 很有吸引力。
(b) 沒有吸引力。

42. 當我在進行一個很長的計算時
(a) 我傾向重複所有步驟並仔細檢查。
(b) 我覺得重複檢查很煩人，必須要強迫自己這麼做。

43. 要想像我曾去過的地方對我來說
(a) 很容易而且大致正確。
(b) 較困難而且不會有太多細節。

44. 當在一個團隊中解決問題時，我更常
(a) 思考解決方案的每一個步驟。
(b) 思考解決方案在更廣泛領域中可能的影響或應用。
